# Intravenous AAV9 administration results in safe and widespread distribution of transgene in the brain of mini-pig

**DOI:** 10.3389/fcell.2022.1115348

**Published:** 2023-01-24

**Authors:** Yingqi Lin, Caijuan Li, Wei Wang, Jiawei Li, Chunhui Huang, Xiao Zheng, Zhaoming Liu, Xichen Song, Yizhi Chen, Jiale Gao, Jianhao Wu, Jiaxi Wu, Zhuchi Tu, Liangxue Lai, Xiao-Jiang Li, Shihua Li, Sen Yan

**Affiliations:** ^1^ Guangdong Key Laboratory of Non-human Primate Research, Guangdong-Hongkong-Macau Institute of CNS Regeneration, Jinan University, Guangzhou, China; ^2^ Key Laboratory of Regenerative Biology, South China Institute for Stem Cell, Biology and Regenerative Medicine, Guangzhou Institutes of Biomedicine and Health, Chinese Academy of Sciences, Guangzhou, China

**Keywords:** AAV9, CNS, large animal, pig, neurodegeneration

## Abstract

Animal models are important for understanding the pathogenesis of human diseases and for developing and testing new drugs. Pigs have been widely used in the research on the cardiovascular, skin barrier, gastrointestinal, and central nervous systems as well as organ transplantation. Recently, pigs also become an attractive large animal model for the study of neurodegenerative diseases because their brains are very similar to human brains in terms of mass, gully pattern, vascularization, and the proportions of the gray and white matters. Although adeno-associated virus type 9 (AAV9) has been widely used to deliver transgenes in the brain, its utilization in large animal models remains to be fully characterized. Here, we report that intravenous injection of AAV9-GFP can lead to widespread expression of transgene in various organs in the pig. Importantly, GFP was highly expressed in various brain regions, especially the striatum, cortex, cerebellum, hippocampus, without detectable inflammatory responses. These results suggest that intravenous AAV9 administration can be used to establish large animal models of neurodegenerative diseases caused by gene mutations and to treat these animal models as well.

## Introduction

Animal models are indispensable in scientific research and often used in the study of disease mechanisms, drug development, and therapeutics. Many animal models of neurodegenerative diseases have been established and thoroughly investigated in the past. These include invertebrate (Lee et al., 2018) and rodent models (Levine et al., 2004; Ashe and Zahs, 2010; Epis et al., 2010; McWilliams et al., 2018), and provide valuable information for understanding how neuropathology and neurological symptoms are developed. However, the genomic homology and complexity (Kwon and Ernst, 2021), the size and structure of the brain (Laramée and Boire, 2015), and the life span (Dutta and Sengupta, 2016) as well as various physiological aspects are noticeably different between small animals and humans, which hampers small animal models in fully modeling the complex pathological features in neurodegenerative diseases, especially in selective neurodegeneration. Therefore, there is an apparent need to establish large animal models that can more closely mimic important pathological and clinical features because of they are more similar to humans in anatomy, physiology and development.

Compared to small animal models, pigs are highly similar to humans in genetics, anatomy, physiology, and neural network complexity (Wernersson et al., 2005; Chen et al., 2007). In addition, the pregnancy period of pigs is short, the number of offspring born in a litter is large, and sexual maturity can be achieved in 5–6 months. These advantages are superior to non-human primates, which normally produce single fetus and have long sexual maturity time with a long pregnancy period. Transgenic pig models for a variety of neurodegenerative diseases have been established, including Alzheimer’s diseases (Kragh et al., 2009; Søndergaard et al., 2012; Jakobsen et al., 2013), Huntington’s disease (Yang et al., 2010; Baxa et al., 2013; Yan et al., 2018), Parkinson’s disease (Yao et al., 2014; Zhou et al., 2015), amyotrophic lateral sclerosis (Chieppa et al., 2014; Yang et al., 2014), spinal muscular atrophy (Lorson et al., 2011), and ataxia–telangiectasia (Kim et al., 2014). Our previous studies have shown that pigs expressing full-length mutant HTT at endogenous levels exhibit neuropathologic and behavioral characteristics similar to the HD patients (Yan et al., 2018). Therefore, pigs have become an attractive large animal model for studying neurodegenerative diseases or other neurological disorders.

Viral vectors are often used to deliver transgenes into the central nervous system to generate neurodegenerative disease models or to treat neurodegenerative diseases. Adeno-associated virus (AAV) is one of the most studied gene therapy tool. So far, multiple AAV serotypes have been characterized, including AAV1-5 and AAV7-9 (Wu et al., 2006). A variety of AAV-based treatments have already been used in clinical research, including Alipogene tiparvovec (Glybera^®^; AMT-011, AAV1-LPLS447X), an adeno-associated virus serotype 1-based gene therapy for adult patients with familial lipoprotein lipase (LPL) deficiency (LPLD) (Scott, 2015), AAV-2 vector for Neurosurgical Delivery of Aspartoacylase Gene (ASPA) to treat Canavan Disease (Janson et al., 2002), a serotype 2 adeno-associated virus expressing CLN2 cDNA for treating late infantile neuronal ceroid lipofuscinos (Worgall et al., 2008), and the voretigene neparvovec gene therapy for patients with RPE65-mediated inherited retinal dystrophy (Russell et al., 2017).

Although AAV has become one of the key tools for preclinical and clinical gene therapy research, how to deliver these AAV carrying the transgenes to the affected brain regions remains a challenge (Mingozzi and High, 2011). To bypass the blood-brain barrier (BBB), intraparenchymal or intrathecal delivery is usually used in current research. However, permanent effects normally cannot be achieved by single AAV administration, and multiple or regular administrations are usually required to produce desired effects (Broekman et al., 2007; Herzog et al., 2007; Worgall et al., 2008; Hudry et al., 2010; Hocquemiller et al., 2016). Although direct injection of AAV into the brain has made great progress, craniotomy still faces many risks and challenges, such as postoperative nausea, vomiting, pain, venous thromboembolism and so on (Anthofer et al., 2016; Fang et al., 2017; Tsaousi et al., 2017). Therefore, there is a need to find an efficient, safe, and simple delivery method of AAVs.

AAV9 is one of the most studied AAV serotypes recently in gene therapy. Compared with other AAV serotypes, AAV9 targets the central nervous system with a higher efficiency and can cross the blood-brain barrier (Duque et al., 2009; Penzes et al., 2021). In addition, AAV9 has a low seropositive rate in the population, which has a significant advantage in gene therapy for humans (Boutin et al., 2010). However, the efficiency of intravenous delivery of adeno-associated virus to the central nervous system in large animals remains to be fully investigated. In the current study, we investigated the efficacy and safety of intravenous delivery of AAV9 in pigs. Our results demonstrated that intravenous administration of AAV9 that expresses GFP did not cause significant inflammatory response and allowed GFP to be widely distributed in the central nervous system in the pigs. These findings provided experimental evidence from large animals for using AAV9 in the preclinical treatment of human brain diseases.

## Results

### Expression of GFP in the central nervous system and peripheral tissues after auricular vein injection of AAV-CMV-GFP in pigs

The purpose of this study is to find an efficient, safe, and simple way of AAV virus delivery to the brain in mini pigs, allowing transgene to cross the blood brain barrier for the gene therapy on neurodegenerative diseases. To this end, AAV-CMV-GFP was injected into mini pigs through the ear vein. The subsequent experiments were then performed to verify the expression of GFP in the pig brain and to explore whether this therapeutic approach yielded any obvious neurotoxicity and inflammatory response.

**FIGURE 1:**
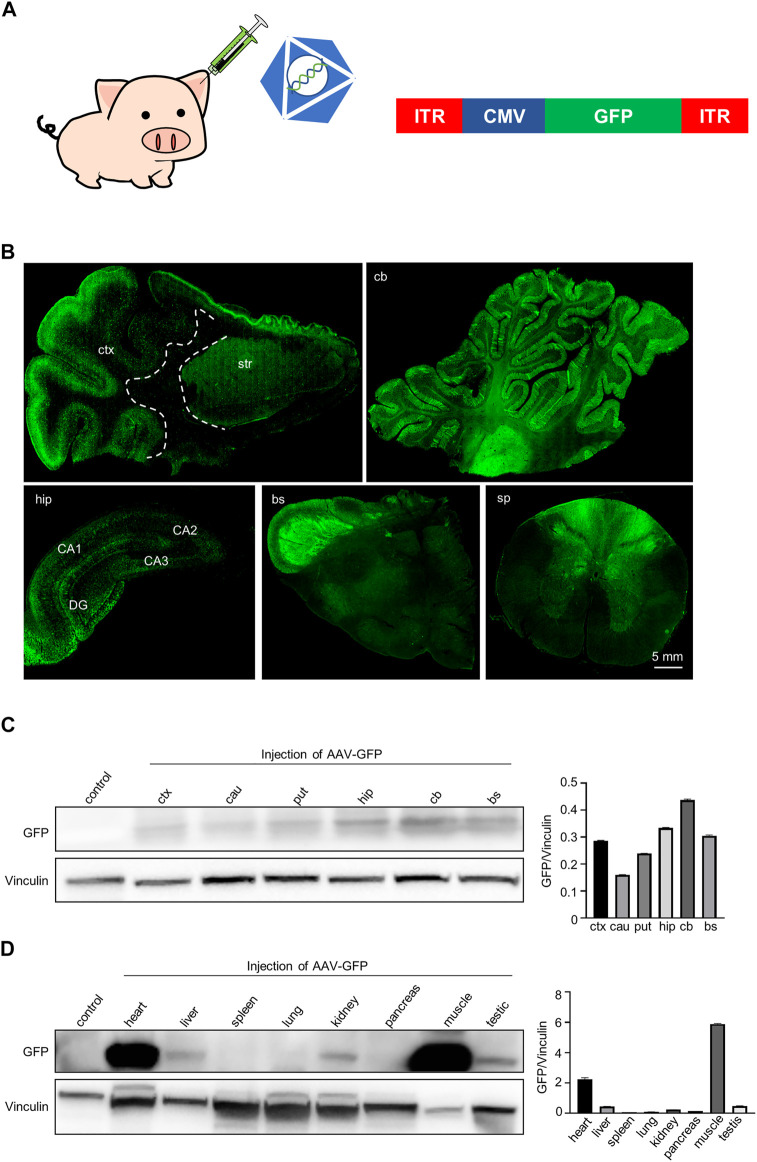
GFP expression in the pig brain, spinal cord and periphery tissues following intravascular administration. **(A)** Injection of AAV-CMV-GFP into 7-day-old Bama pigs *via* auricular vein. Saline served as a control. AAV9 serotype was used to package viruses. **(B)** Immunofluorescent staining showed that GFP was expressed in various brain regions and spinal cord of pigs after AAV-CMV-GFP injection. Regions are (clockwise from upper left) cortex and striatum (ctx and str), cerebellum (cb), hippocampus (hip), brain stem (bs), spinal cord (sp). **(C)** Western blotting demonstrates that GFP was expressed in various brain regions of pigs. Vinculin served as a loading control (left). Quantification of the GFP expression in each brain region. Data are analyzed by Student’s T-test and presented as mean ± SEM (right). N = 3 animals per group. **(D)** Western blotting of GFP expression in various peripheral tissues of pigs. Vinculin served as a loading control (left). Quantification of GFP expression in each type of peripheral tissues. Data are analyzed by Student’s T-test and presented as mean ± SEM (right). N = 3 animals per group.

As a disease model, pigs have been widely used in the study of central nervous system diseases. AAV, as a delivery tool for foreign genes, is also widely used in the study of central nervous system diseases. But how to safely and efficiently deliver AAV to the central nerve system of pigs has not been studied. Because the blood-brain barrier of newborn piglets is not fully established, AAV-9 virus has been proved that it can cross the blood-brain barrier relatively easily and can be distributed widely in the brain after intravenous injection of newborn pigs (Foust et al., 2009). Therefore, we injected the AAV-CMV-GFP through auricular vein into 7-day-old piglets at a dose of 1.2 × 10^13^ genome copies/kg (Figure 1A). GFP was expressed by AAV9 vector under the control of the CMV promoter (AAV-CMV-GFP). This virus can be widely expressed in a variety of cell types and was selected for intravenous injection such that GFP represents transgene expression that can be readily detected.

We first evaluated the expression of GFP in the mini-pig. We detected the expression of GFP in the central nervous system, including the cortex, striatum, cerebellum, hippocampus and spinal cord by using immunofluorescent staining with anti-GFP antibody (Figure 1B). GFP-positive cells were widely distributed in the cortex and were more abundant in the gray matter region where neuronal cells are enriched. GFP-positive cells were also frequently seen in the striatum (Figure 1B, top left panels). The cerebellum contained abundant GFP-positive neurons and glia cells, and most Purkinje cells expressed GFP (Figure 1B, top right panels). We also observed a large number of GFP-positive cells in the hippocampus (Figure 1B, bottom right panel). Many GFP-positive fibers were observed mainly in the brainstem and spinal cord, potentially reflecting fibers in afferent and efferent brain regions (Figure 1B, bottom, center, right panel). Consistently, western blotting results showed that GFP was wildly expressed in the brain regions, including the cortex, caudate, putamen, cerebellum and hippocampus (Figure 1C). In addition, GFP was also expressed in the peripheral tissues of pig, especially in heart and muscle. Interestingly, we found that GFP was expressed in the testis, which means that the virus can enter the seminiferous ducts through the blood-testis barrier when being injected through the auricular vein in newborn animals (Figure 1D). Taken together, a single intravenous injection of AAV-CMV-GFP into the newborn pigs allowed GFP expression throughout the pig body.

### Effects of AAV-GFP transduction on the expression of neuronal and glial proteins

**FIGURE 2:**
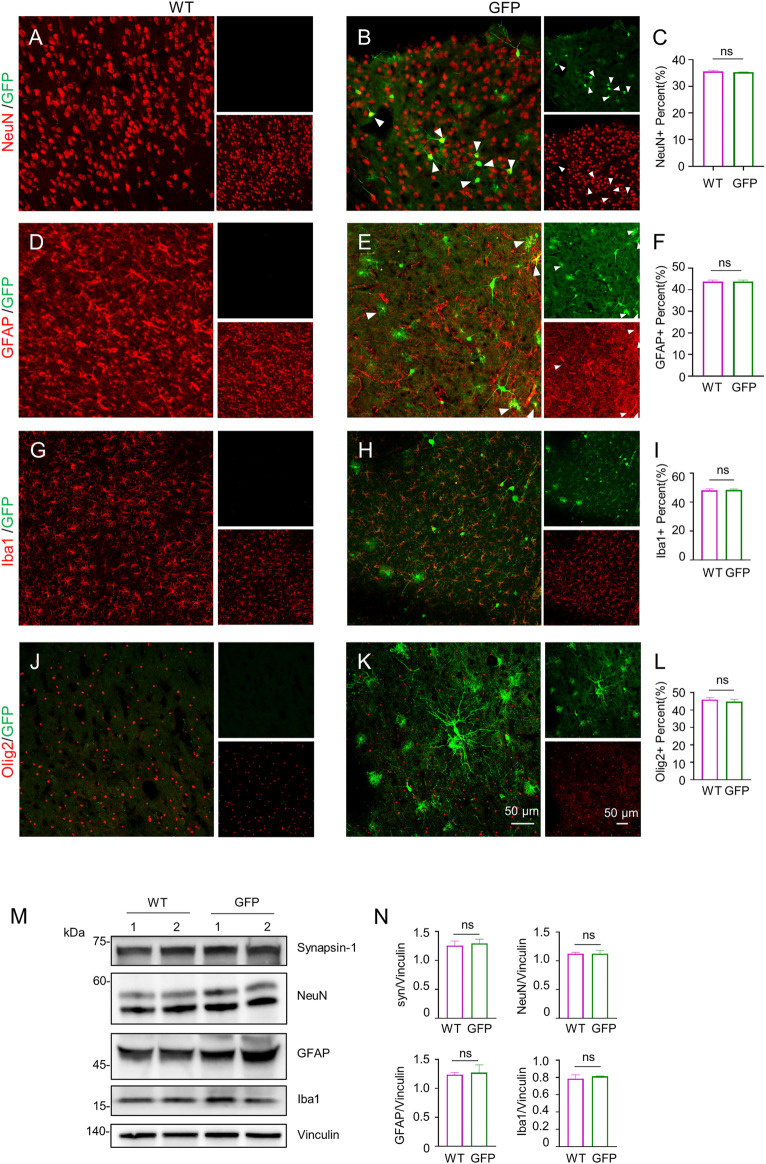
Immunofluorescent staining of the pig’s cortex injected with AAV-GFP or saline. **(A–C)** Double immunofluorescent labeling **(A,B)** and quantification **(C)** of neurons (NeuN) of saline- or AAV-GFP-injected-wild type pigs. GFP positive cells are shown in green (small images in the upper right), NeuN positive cells are shown in red (small images in the lower right). The triangles indicate cells that are positive for both GFP and NeuN. **(D–F)** Double-immunofluorescent labeling **(D,E)** and quantification **(F)** of astrocytes (GFAP) in saline control or AAV-GFP injected pigs. Small images in the upper right are GFP positive cells and in the lower right are GFAP positive cells. **(G–I)** Double-immunofluorescent labeling **(G,H)** and quantification **(I)** of microglial cells (Iba1) in saline control or AAV-GFP injected pigs. Small images in the upper right are GFP positive cells and in the lower right are Iba1 positive cells. **(J–L)** Double-immunofluorescent labeling **(G,H)** and quantification **(L)** of oligodendrocytes (Olig2) cells in the saline control or AAV-GFP injected pigs. Small images in the upper right are GFP positive cells and in the lower right are Olig2 positive cells. Quantification of the numbers of neuronal or glial cells was performed using three animals per group. Data are analyzed by Student’s T-test and presented as mean ± SEM. **(M)** Western blotting of the cortex of the saline- or AAV-GFP-injected pigs with antibodies against synapsin-1, NeuN, GFAP, and Iba1. Vinculin served as a loading control. **(N)** Quantitation of the ratios of synapsin-1, NeuN, GFAP and Iba1 to vinculin on the western blots. Data are analyzed by student’s T-test and presented as mean ± SEM. *n* = 3 animals per group.

**FIGURE 3:**
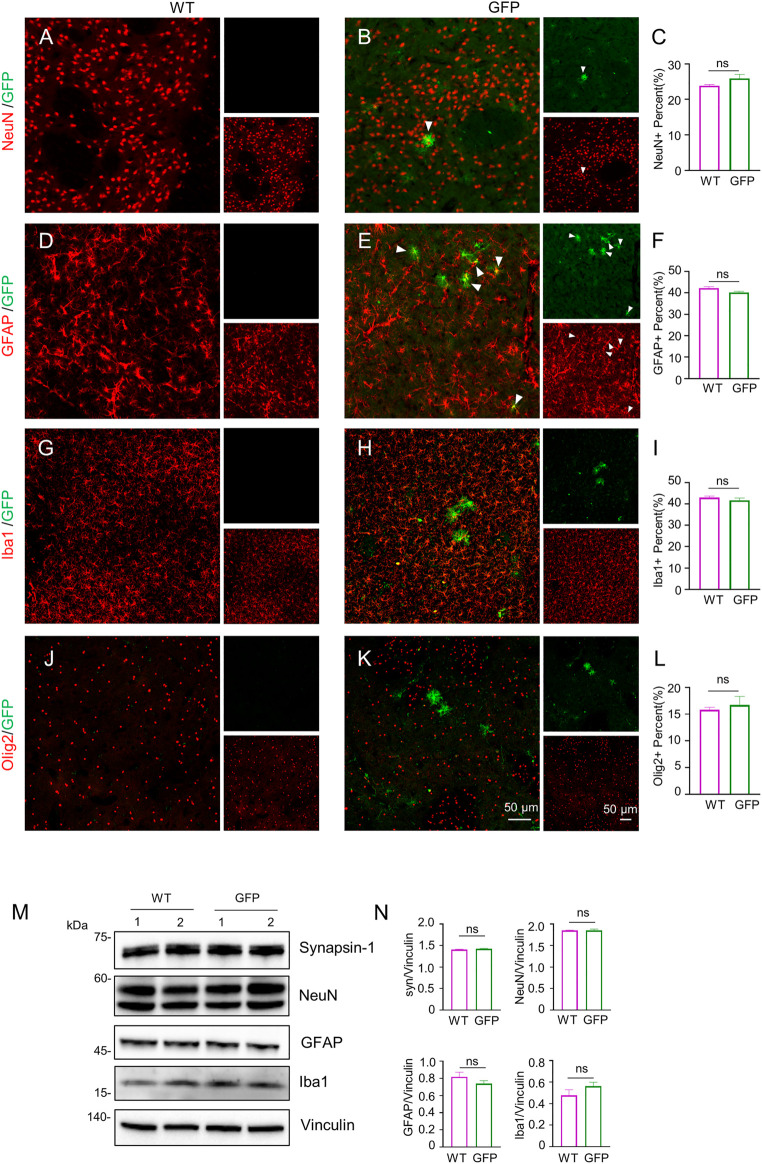
Immunofluorescent staining of the pig’s striatum injected with AAV-GFP or saline. **(A–L)** Double immunofluorescent labeling and quantification of neurons (NeuN) **(A–C)**, astrocytes (GFAP) **(D–F)**, microglial (Iba1) **(G–I)** and oligodendrocytes (Olig2) **(J–L)** of saline- or AAV-GFP-injected-wild type pigs. GFP positive cells are shown in green, NeuN, GFAP, Iba1 and Olig2 positive cells are shown in red. Data are analyzed by Student’s T-test and presented as mean ± SEM. *n* = 3 animals per group. **(M)** Western blotting of the striatum of saline- or AAV-GFP-injected pigs with antibodies against synapsin-1, NeuN, GFAP and Iba1. Vinculin served as a loading control. **(N)** Quantitation of the ratios of synapsin-1, NeuN, GFAP or Iba1 to vinculin on the western blots. Data are analyzed by student’s T-test and presented as mean ± SEM. *n* = 3 animals per group.

**FIGURE 4:**
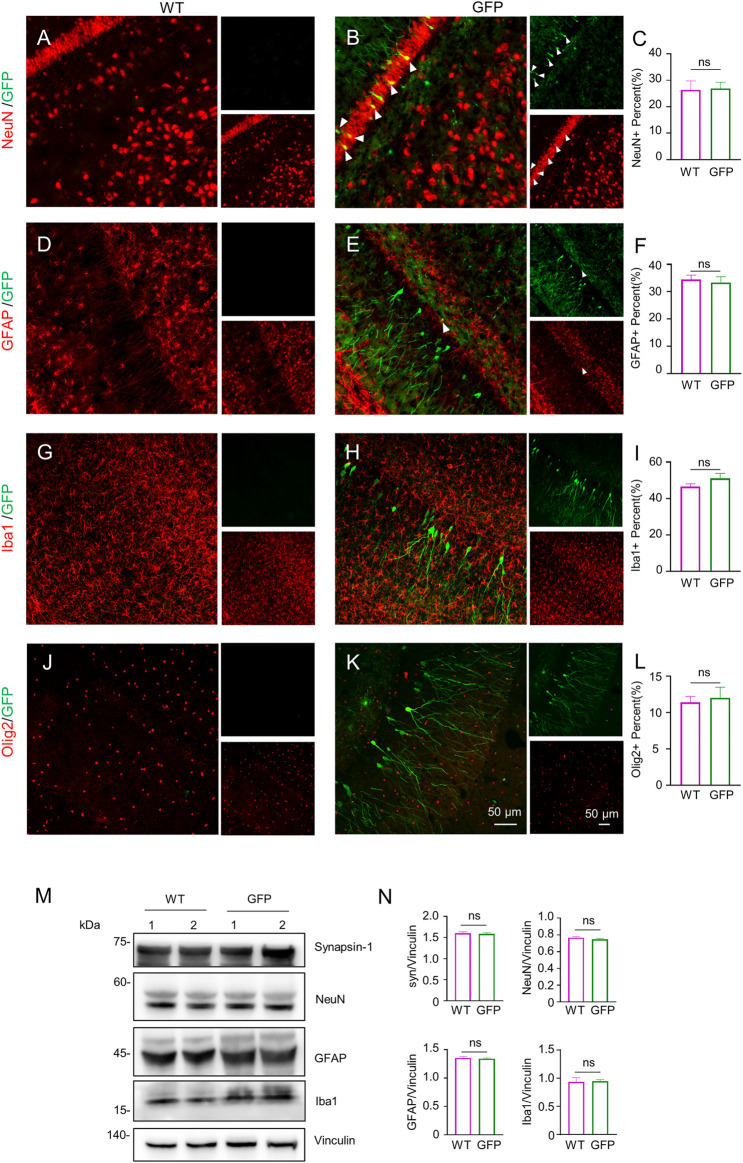
Immunofluorescent staining of the pigs’ hippocampus injected with AAV-GFP or saline. **(A–L)** Double immunofluorescent labeling and quantification of neurons (NeuN) **(A–C)**, astrocytes (GFAP) **(D–F)**, microglial (Iba1) **(G–I)** and oligodendrocytes (Olig2) **(J–L)** of saline- or AAV-GFP-injected-wild type pigs. GFP positive cells are shown in green, NeuN, GFAP, Iba1 and Olig2 positive cells are shown in red. Data are analyzed by Student’s T-test and presented as mean ± SEM. *n* = 3 animals per group. **(M)** Western blotting of the hippocampus of saline- or AAV-GFP-injected pigs with antibodies against synapsin-1, NeuN, GFAP and Iba1. Vinculin served as a loading control. **(N)** Quantitation of the ratios of synapsin-1, NeuN, GFAP or Iba1 to vinculin on the western blots. Data are analyzed by student’s T-test and presented as mean ± SEM. *n* = 3 animals per group.

**FIGURE 5:**
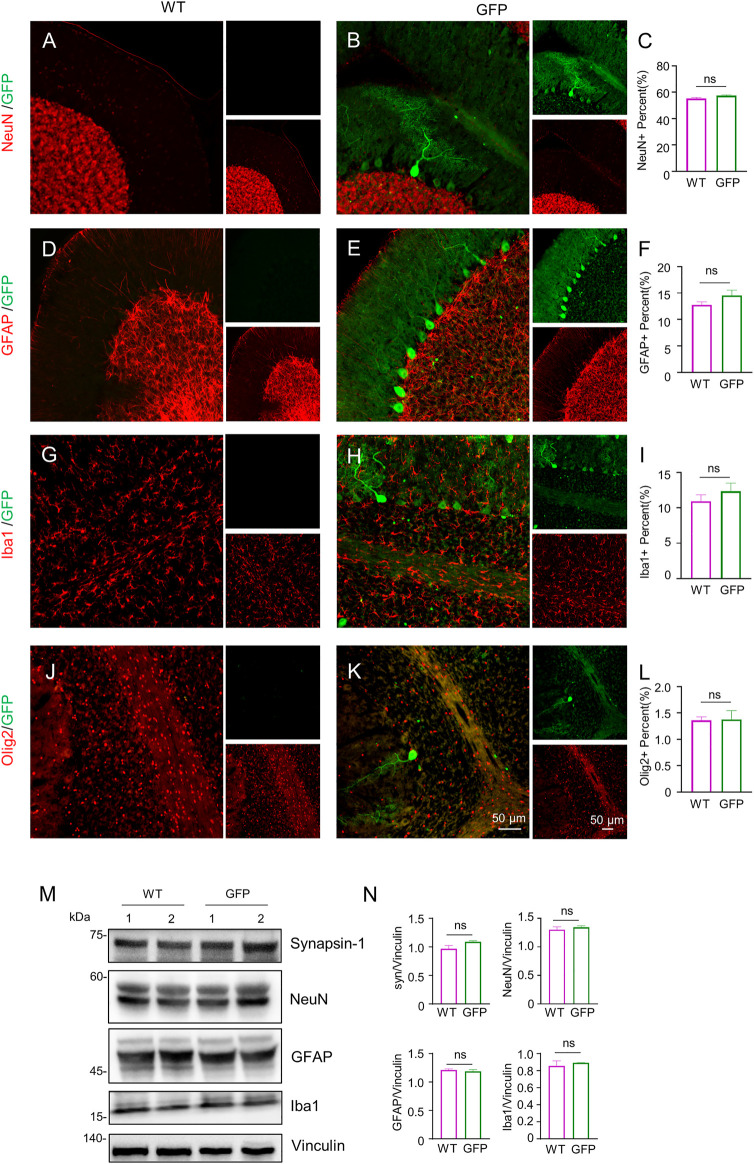
Immunofluorescent staining of the pigs’ cerebellum injected with AAV-GFP or saline. **(A–L)** Double immunofluorescent labeling and quantification of neurons (NeuN) **(A–C)**, astrocytes (GFAP) **(D–F)**, microglial (Iba1) **(G–I)** and oligodendrocytes (Olig2) **(J–L)** of saline- or AAV-GFP-injected-wild type pigs. GFP positive cells are shown in green, NeuN, GFAP, Iba1 and Olig2 positive cells are shown in red. Data are analyzed by Student’s T-test and presented as mean ± SEM. *n* = 3 animals per group. **(M)** Western blotting of the cerebellum injected with saline or AAV-GFP using antibodies against synapsin-1, NeuN, GFAP and Iba1. Vinculin served as a loading control. **(N)** Quantitation of the ratios of synapsin-1, NeuN, GFAP or Iba1 to vinculin on the western blots. Data are analyzed by student’s T-test and presented as mean ± SEM. *n* = 3 animals per group.

To assess the effect of AAV-GFP transduction on neuronal and glial cells, we used antibodies against neuronal protein (NeuN) for detecting neurons (Gusel’nikova and Korzhevskiy, 2015), glial fibrillary acidic protein (GFAP) for astrocytes (Baba et al., 1997; Eng et al., 2000; Li et al., 2020), ionized calcium binding adapter molecule 1 (Iba1) for microglia (Ito et al., 1998; Sasaki et al., 2001), and oligodendrocyte lineage transcription factor 2 (Olig2) for oligodendrocytes (Zhou et al., 2000). The number s of neurons, astrocytes, microglia and oligodendrocytes in the cortex of wild type (WT) pigs injected with virus did not change, compared with WT pigs injected with saline. (Figures 2A–L). Similarly, the results of western blotting demonstrated that synapsin-1, which is involved in regulation of neurotransmitter release, NeuN, GFAP and Iba1 were unchanged in cortex (Figure 2M, N). In brain regions where numerous cell bodies were also GFP-labeled, including the striatum (Figure 3), hippocampus (Figure 4) and cerebellum (Figure 5), cell numbers and synapsin-1 staining did not change, consistent with the results observed in the cortex. Similarly, there were also no changes in the above-mentioned neuropathology-related markers in the brain stem (Supplementary Figure S1) and spinal cord (Supplementary Figure S2), though GFP-tagged fibers were present in these two regions. We then compared GFP expression in the cortex (Figure 2), striatum (Figure 3), and hippocampus (Figure 4) using double immunohistochemical staining and found that AAV-CMV-GFP was more abundant in neurons (Figure 2B; Figure 3B; Figure 4B) and astrocytes (Figure 2E; Figure 3E; Figure 4E) than microglia (Figure 2H; Figure 3H; Figure 4H) and oligodendrocytes (Figure 2K; Figure 3K; Figure 4K). A qualitative analysis of transduction levels of each cell type is in Supplementary Table S1. What is strikingly noticeable is that in the cerebellum the virus basically infected neurons, especially Purkinje cells, and did not infect glial cells (Figure 5). Taken together, injecting the AAV-CMV-GFP into pigs through auricular vein did not cause neuronal damage and glial cell activation, and the virus infected basically neurons and astrocytes.

### AAV-CMV-GFP injection in pigs *via* auricular vein does not cause inflammatory response

**FIGURE 6:**
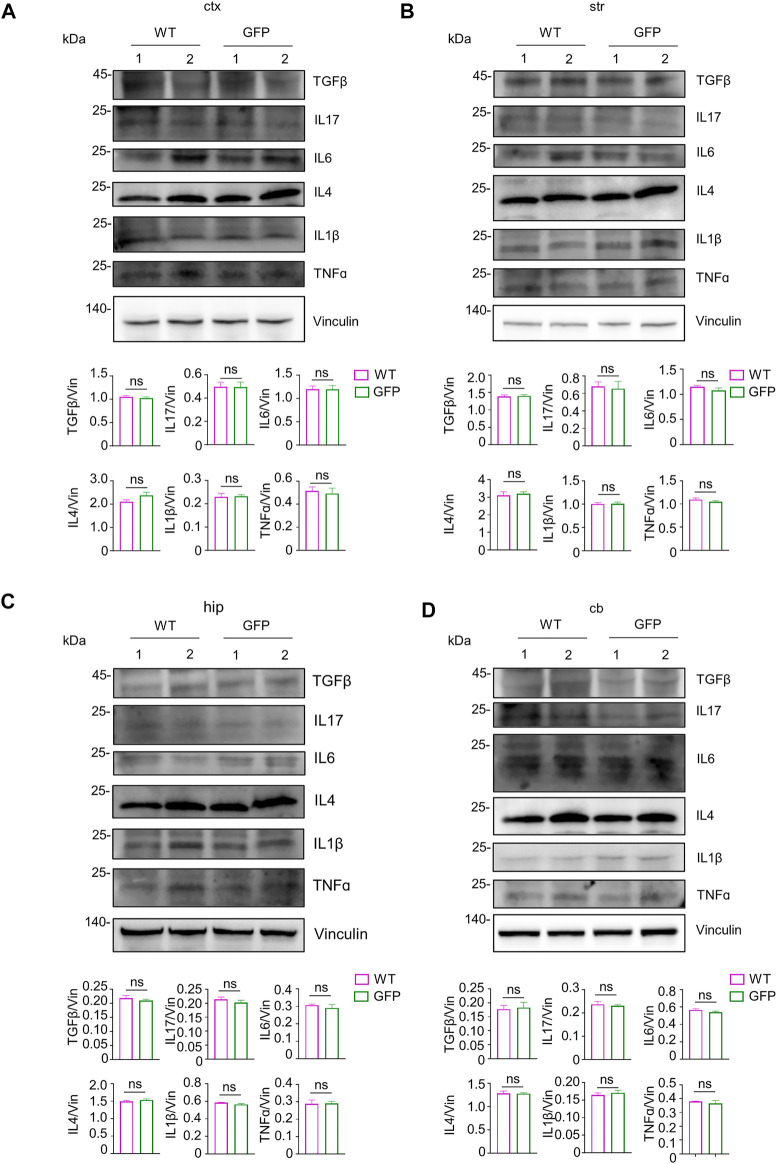
Examination of inflammatory factors in the pig’s central nervous system after intravenous injection of saline or AAV-GFP. **(A–D)** Western blotting of the cortical tissue **(A)**, striatum **(B)**, hippocampus **(C)** and cerebellum **(D)** of saline-or AAV GFP-injected pigs with antibodies against TGFβ, IL17, IL6, IL4, IL1β and TNFɑ. Vinculin served as a loading control. Quantitation of the ratios of TGFβ, IL17, IL6, IL4, IL1β and TNFɑ to vinculin on the western blots are presented beneath the blots. Data are analyzed by Student’s T-test and presented as mean ± SEM. *n* = 3 animals per group.

Inflammation within the central nervous system can damage neurons (Herz et al., 2010; Ashraf et al., 2021). In addition, the degree of inflammatory response in the central nervous system is associated with systemic inflammation. Inhibiting the inflammatory response in the peripheral tissues can improve inflammation in the central nervous system (Campbell et al., 2007, 2008; Anthony and Couch, 2014; Clausen et al., 2014). To test the safety of intravenous administration of AAV, we next investigated whether intravenous injection of AAV-CMV-GFP could cause inflammatory responses in the central nervous system and peripheral tissues. Because the expression of inflammatory cytokines is a pathological feature of inflammation in the brain, spinal cord, and periphery (Zhang and An, 2007; Becher et al., 2017), we chose to examine the expression of several typical inflammatory cytokines, including TGFβ, IL17, IL6, IL4, IL1β, TNFɑ, in the AAV-GFP-injected pigs and compare them with those in the saline-injected pigs. Western blotting results showed no significant changes in the expression of inflammatory cytokines detected in the cortex of pigs after virus injection (Figure 6A). Similar, similar results were observed in the striatum (Figure 6B), hippocampus (Figure 6C), cerebellum (Figure 6D), and brain stem (Supplementary Figure S3A). The expression of these inflammatory cytokines was also largely unchanged in the spinal cord (Supplementary Figure S3B) and peripheral tissues (Supplementary Figure S4). The above results indicate that intravenous injection of AAV-CMV-GFP is safe and does not cause severe inflammatory reactions in any major organ.

### AAV-CMV-GFP injection in pigs *via* auricular vein does not cause transcriptome alternation

**FIGURE 7:**
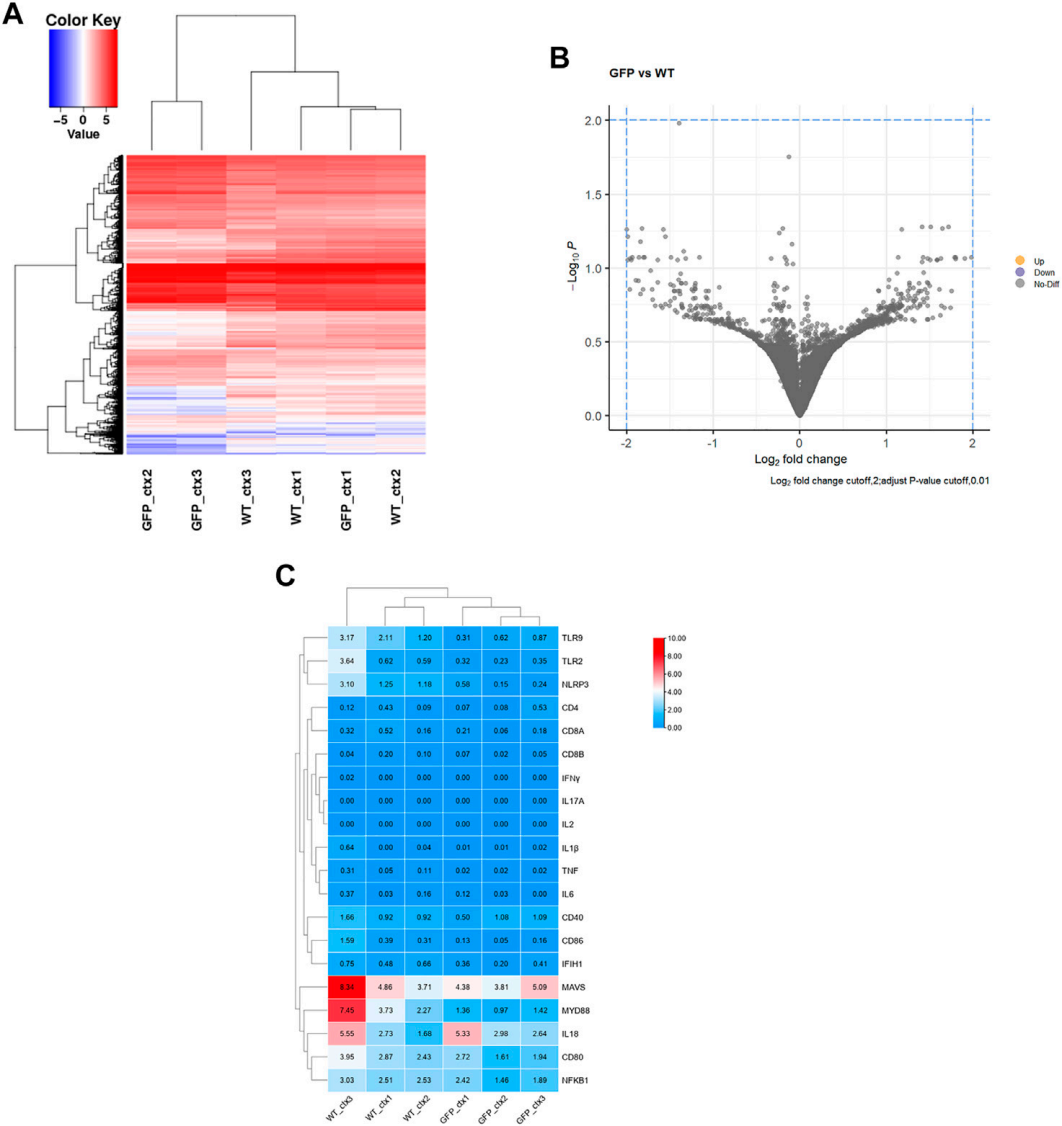
Transcriptome analysis of the intravenous AAV9-injected pig cortical tissues. **(A)** Heatmap of differential gene expression, blue and red color intensities represent gene downregulation and upregulation, respectively. **(B)** GFP vs. WT differential gene volcano plot, Log_2_ fold change cutoff,2; adjust *p*-value cutoff,0.01. **(C)** Heatmap of expression of 20 common genes associated with inflammatory responses.

To further determine the effect of intravenous AAV9 on the central nervous system, we performed RNA-seq analysis of the cortical tissues, which has high GFP expression and contains major part of the brain. The results showed that the transcriptomes of in the cortex of pigs receiving intravenous AAV-CMV-GFP (GFP) were very similar to those of pigs receiving saline treatment (WT) (Figure 7A; Supplementary Figure S5). Further analysis of the differential expression at the same difference threshold (log2FC > 2, Padj<.01) revealed no significant difference between WT and AAV-GFP groups (Figure 7B), though slight but not significant differences may exist. We also examined 20 genes associated with inflammation between WT and GFP groups and found no significant changes in these genes (Figure 7C), suggesting that intravenous administration of AAV9 in large animals did not induce a strong inflammatory response. RNA-seq showed that intravenous administration of AAV9 did not cause changes in the transcriptional regulation or inflammatory response in the cortical tissues of pigs injected with AAV-GFP.

## Discussion

Intravenous delivery of AAV9 to target specific genes in the central nervous system has great therapeutic potential for a variety of neurodegenerative diseases, such as Alzheimer’s, Huntington’s, Parkinson’s diseases, amyotrophic lateral sclerosis, spinal muscular atrophy, ataxia telangiectasia *etc.* Currently, not many studies have evaluated the transduction efficiency, distribution pattern, and safety of AAV9 after direct intravenous delivery in the central nervous system of large animals such as pigs. Our results demonstrated the ability of AAV9 to transduce neurons and glial cells in various brain regions and spinal cord of pigs by injecting AAV-CMV-GFP into the auricular vein.

Many neurodegenerative diseases often start with selective specific brain regional cell death (Double et al., 2010; Reith, 2018), and as diseases progress, neurodegeneration becomes more severe and extends to other brain regions. Brain regional administration can be used to treat brain-region specific damage but would be difficult to reach to the large area of brain parenchyma. However, AAV9 can spreading to various brain regions and is able to pass blood brain barrier, providing a possibility for the treatment of disease that affects large areas in brain.

In our study, neuronal cells in both the cortex and striatum were found to express transgene GFP after intravenous injection of AAV-CMV-GFP. The widespread expression of transgene in these two brain regions is important for treating Huntington disease (HD), which is caused by polyglutamine repeat expansion in the Huntingtin (HTT) protein and display neurodegeneration that is most severe in the striatum and cortex (Bates et al., 2015). In both HD patients and HD KI pig models, the medium spiny neurons in the striatum undergo preferential neurodegeneration. With the development of the disease, the cortex and other brain regions will also be involved (Waldvogel et al., 2015; Yan et al., 2018). Because HD is caused by a single gene mutation, using gene therapy to inhibit the expression of mutated HTT is one of the attractive strategies to treat HD. Previous study has used HDKI-140Q mice to test gene therapy because they express mutant HTT in the same manner as patients with HD. However, rodents cannot truly mimic the neuropathology seen in HD patients, and HD KI mice expressing full-length mutant HTT at endogenous level lack significant pathological features of neuronal loss (Levine et al., 2004; Yang et al., 2017). On the other hand, HD KI pigs we established previously show striking neuronal loss as HD patients (Yan et al., 2018), providing a valuable model to evaluate the effects of gene therapy on neurodegeneration. Thus, it is important to use pigs as a model to examine whether AAV administration can effectively deliver transgene into the pig brain.

Previous studies have used intracranial or intratarsal injection of AAV into the pig brain to suppress HTT expression (Evers et al., 2018; Vallès et al., 2021). It remains unknown whether a single intravenous injection of AAV into large animals can lead to the broad distribution of transgene in various brain regions. We used AAV9-GFP for intravenous injection in pigs so that the expression of transgene (GFP) could be identified in the pig brain. In addition to the striatum and cortex that are most affected in HD, GFP was expressed in the hippocampus, cerebellum (especially in Purkinje cells), brainstem, and spinal cord. These brain regions have been reported to be affected in a number of neurodegenerative diseases. For example, in patients with Alzheimer’s disease, the abnormal accumulation of Aβ plaques and Tau tangles leads to neuronal cell death in the entorhinal cortex and hippocampus first, with plaques and tangles gradually being spread in the frontal lobe, parietal lobe, globus pallidus and other brain regions (Čaušević et al., 2010; Latimer et al., 2019). A common and fatal heritable spinal muscular atrophy in infants is caused by the loss of alpha motor neurons in the spinal cord (Crawford and Pardo, 1996), and amyotrophic lateral sclerosis (ALS) is a fatal neurodegenerative disease with the degeneration of motor neurons in the brain stem of the motor cortex and spinal cord (Wang et al., 2015; Bonafede and Mariotti, 2017). SCA3 is caused by an abnormal CAG repeat expansion in exon 10 of the ATXN3 gene with progressive motor and neuronal dysfunction, in the somatosensory and motor nuclei spanning brainstem, cerebellum, midbrain, spinal cord, striatum, and thalamus (McLoughlin et al., 2020). For all the brain disordered in the above-mentioned classes, intravenous injection has an advantage in the treatment, and once AAV9 passes through the blood-brain barrier, the transgene can be expressed in the specific populations of cells or selective brain regions under the control of specific promoters.

It should be point out that intravenous AAV delivery can induce immune and inflammatory responses, which would also be dependent on animal size, brain anatomy, and physiology in different species. Our findings demonstrated the efficacy and safety of intravenous injection of AAV-CMV-GFP in transducing the central nervous system of piglet. Currently, lipid nanoparticles (LNPS) have become an attractive treatment tool due to their low immunogenicity, low production costs, and the capability to deliver various goods. However, LNPS tend to target peripheral tissue, as they are difficult to pass the blood brain barrier to deliver the cargoes to the brain, and do not yield long-term transgene expression. Therefore, the gene expression of AAV is currently a more effective treatment for neurodegenerative diseases.

We found that a single intravenous injection of AAV-CMV-GFP can lead to a widespread expression of GFP in the central nervous system in a large animal model. Because the sizes of brain and body of pigs are similar to those of humans, the non-invasive nature of intravenous injection of AAV and widespread expression of transgene in pigs reinforce the therapeutic potential of intravenous injection of AAV to treat neurological disorders in humans.

## Materials and methods

### Animals and ethics statement

Bama pigs are local strains from Southern China. The pigs were breed at the animal Facility of the Guangzhou Institute of Biomedicine and Health (GIBH), Chinese Academy of Sciences (Animal Welfare Assurance #N2019083). Animal use and care followed the NIH Guide for the Care and Use of Laboratory Animals. The Institutional Animal Care and Use Committees (IACUC) at Guangzhou Institute of Biomedicine and Health (GIBH), Chinese Academy of Sciences approved the animal use protocol. This study carried out in strict compliance with the “Guide for the Care and Use of Laboratory Animals (2011)” to ensure the safety of personnel and animal welfare. The pigs used in the current experiments were wild-type pigs, and maintained under in-door housing conditions at room temperature in the Animal Center of Guangzhou Institutes of Biomedicine and Health. Regular food and water were provided *ad libitum*.

### Virus production and injection

GFP-expressing viral vector was obtained from Addgene (plasmid# 67634). The vector was packaged by PackGene Biotech with the AAV9 serotype. Purified viruses were stored at -80°C. The genomic titer of the purified viruses (vg) (approximate 10^13^ vg/mL) was determined by PCR method.

The same litter of 7-day-old Bama pigs were injected with AAV-CMV-GFP or saline as the experimental group or the control group. The pigs were anesthetized with 1.5% isoflurane, and the surgical site was sterilized with a betadine solution (10% povidone-iodine) followed by 75% ethanol. A 30G needle attached to a 1 ml Hamilton syringe was inserted into the auricular vein. Viruses (300 ml of 10^13^ vg/ml diluted in 1 ml saline) or the same volume of saline were injected through the auricular vein into each pig over the course of 5 min. After the infusion is complete, the needle was left in place for 3 min and then slowly removed from the pigs. Pigs were placed on a warm cuddy after surgery to allow them to recover from anesthesia. After the piglets woke up, they were sent back to their mothers.

### Necropsy and tissue collection

Two months after the virus injection, the pigs were euthanized by deep anesthesia with intraperitoneal injection of .3–.5 ml of atropine, followed by 10–12 mg of ketamine per kg body weight and perfused with 3 L of a sterile .9% sodium chloride solution through the left ventricle of the heart. The brain was removed from the skull, the left hemisphere was immediately frozen on dry ice after the brain regions were separated, and the right hemisphere was emersed in 10 volumes of 4% paraformaldehyde (PFA) for fixation for 48 h.

### Western blot analysis and immunohistochemistry

The primary antibodies against the following proteins were used in the study: synapsin-1 (Cell Signaling, 5297 S), NeuN (Abcam, ab177487), GFAP (Abcam, ab7260), IBA1 (WAKO, 019-19741), Olig2 (Milipore, MABN50), GFP (Invitrogen, A11122), TGFβ (Abcam, ab215715), IL17 (Santa Cruz, sc-374218), IL6 (abcam, ab6672), IL4 (Proteintech, 66142-1-Ig), IL1β (abcam, ab9722), TNF (abcam, ab1793). Secondary antibodies were all from Jackson ImmunoResearch laboratories, INC, Abcam and Invitrogen.

For western blot analysis, pig brain tissues were lysed in ice-cold RIPA buffer (50 mmol/L Tris, pH 8.0, 150 mmol/L NaCl, 1 mmol/L EDTA pH 8.0, 1 mmol/L EGTA pH 8.0, .1% SDS, .5% DOC, 50 mmol/L NaF and 1% Triton X-100) containing Halt protease inhibitor cocktail (Thermo Scientific) and PMSF (Sigma). The pig brain tissues were grinded by using a Luka Grinding instrument (LUKYM-II, China) and the tissue lysates were incubated on ice for 30 min, centrifuged at 12,000 rpm for 10 min. Protein concentrations in the supernatants were determined by BCA assay (Bio-Rad), and equal amount of proteins were loaded to SDS-PAGE and subsequently transferred to a nitrocellulose membrane. The membrane was blocked with 5% milk/TBST for 1 h at room temperature. Primary antibodies were diluted in 3% BSA/TBST and incubated with the membrane overnight at 4°C. The membranes were then washed 3 times with TBST and incubated with HRP-conjugated secondary antibodies in 5% milk/TBST for 1 h at room temperature. After washing with TBST, the signals on the membrane were detected with the ECL Prime (GE Healthcare) kit.

For immunofluorescent study, the isolated pig brain tissues were fixed for 48 h in 4% paraformaldehyde/0.01 M PBS and then transferred into 30% sucrose to dehydrate at 4°C until the brain completely sank to the bottom of the tube. The tissues embedded in Tissue-Tek^®^O.C.T.Compound (Tissue-Tek, Sakura Finetek) and frozen in liquid nitrogen with an isopentane interphase. The consecutive pig brain coronal sections of 30 μm were cut with freezing microtome. The pig tissue slides were fixed in 4% paraformaldehyde in .01 M PB for 10 min, and pre-blocked with 4% normal goat serum in .1% Triton X-100/PBS for 1 h. Slides were incubated with primary antibodies in 3% BSA/2%NGS/TBST overnight at 4°C. Secondary antibodies were added after three washes with PBS. Microscopic images were acquired by TissueFAXS PLUS (TissueGnostics, Vienna, AUT) and a confocal imaging system ((Olympus FV3000 Microscope).

### RNA-seq and data analysis

Total RNA of the cortex in WT pigs injected with saline or WT pigs injected with AAV-CMV-GFP through the auricular vein were isolated using RNAiso Plus (TaKaRa). The RNAs were sent to HeQin Biotechnology Corporation (Guangzhou) for RNA-seq analysis and database construction. A total of 2 mg of RNAs per sample were used for analysis. NEBNext Ultra RNA Library Prep Kit for Illumina (E7530L; NEB) was used for sequencing according to the manufacturer’s recommendations. After cluster generation, the libraries were sequenced and 150-bp paired-end reads were generated using Illumina platform. After obtaining the raw sequencing data, Trimmomatic software was used to control the quality of raw RNA-seq data and remove the sequencing adapter (Bolger et al., 2014). We then used STAR software (STAR: ultrafast universal RNA-seq aligner - PMC) to map the clean data to the pig genome, which was downloaded from the Ensembl website (Howe et al., 2020), to obtain the sam files. The samtools (Li et al., 2009) was used to convert sam files into bam files, sort and build index files. We used stringtie (Pertea et al., 2015) and its script ‘prepDE.py’ to quantify genes and convert them into read counts matrix. Finally, the R package DESeq2 (Love et al., 2014) was used for gene differential expression analysis, and the read counts matrix was used as the input file. Genes with adjusted *p*-value <.01 and an absolute fold change >2 were considered as DEGs. GO enrichment analysis for DEGs in a group was carried out using TBtools (Chen et al., 2020). GO terms with a *p*-value <.01 and a hit rate >.05 were considered significantly enriched. In addition, PCA analysis and heat map were also performed using carrieUBC. The RNAseq data have been deposited with the GEO number PRJNA911021.

### Statistical analysis

When every two groups were compared, statistical significance was assessed with the two-tailed Student’s t-test. Data are presented as mean ± SEM. For pathological examination, western blotting, and RNA-seq, at least three animals per group were used. Calculations were performed with GraphPad Prism software (GraphPad Software). A *p*-value of .05 was considered statistically significant.

## Data Availability

The data presented in the study are deposited in the NCBI Trace Archive NCBI SequenceRead Archive repository, accession number PRJNA911021.

## Supplementary material

Supplementary Material S1 Three brain regions were chosen (cortex, striatum and hippocampus) for qualitative rating of cell type transduction. Scale ranges from − to ++++, with − indicating no double labeling of GFP and the cell specific marker and ++++ indicating the highest level of double labeling observed.

Supplementary Material S1 Three brain regions were chosen (cortex, striatum and hippocampus) for qualitative rating of cell type transduction. Scale ranges from − to ++++, with − indicating no double labeling of GFP and the cell specific marker and ++++ indicating the highest level of double labeling observed.

Supplementary Figure S1 Immunofluorescent staining of the pig’s brain stem injected with AAV-GFP or saline. **(A–L)** Double immunofluorescent labeling and quantification of neurons (NeuN) **(A–C)**, astrocytes (GFAP) **(D–F)**, microglial (Iba1) **(G–I)** and oligodendrocytes (Olig2) **(J–L)** of saline- or AAV-GFP-injected-wild type pigs. GFP positive cells are shown in green, NeuN, GFAP, Iba1 and Olig2 positive cells are shown in red. Data are analyzed by Student’s T-test and presented as mean ± SEM. n = 3 animals per group. **(N)** Western blotting of the brain stem of saline- or AAV-GFP-injected pigs with antibodies against synapsin-1, NeuN, GFAP and Iba1. Vinculin served as a loading control. **(N)** Quantitation of the ratios of synapsin-1, NeuN, GFAP or Iba1 to vinculin on the western blots. Data are analyzed by student’s T-test and presented as mean ± SEM. n = 3 animals per group.

Supplementary Figure S2 Immunofluorescent staining of the pig’s spinal cord injected with AAV-GFP or saline. **(A–L)** Double immunofluorescent labeling and quantification of neurons (NeuN) **(A–C)**, astrocytes (GFAP) **(D–F)**, microglial (Iba1) **(G–I)** and oligodendrocytes (Olig2) **(J–L)** of saline- or AAV-GFP-injected-wild type pigs. GFP positive cells are shown in green, NeuN, GFAP, Iba1 and Olig2 positive cells are shown in red. Data are analyzed by Student’s T-test and presented as mean ± SEM. n = 3 animals per group. **(M)** Western blotting of the spinal cord of saline- or AAV-GFP-injected pigs with antibodies against synapsin-1, NeuN, GFAP and Iba1. Vinculin served as a loading control. **(N)** Quantitation of the ratios of synapsin-1, NeuN, GFAP or Iba1 to vinculin on the western blots. Data are analyzed by student’s T-test and presented as mean ± SEM. n = 3 animals per group.

Supplementary Figure S3 Examination of inflammatory factors in the pig’s brain stem and spinal cord after intravenous injection of saline or AAV-GFP. **(A,B)** Western blotting of the brain stem **(A)** and spinal cord **(B)** of saline-or AAV GFP-injected pigs with antibodies against TGFβ, IL17, IL6, IL4, IL1β and TNFα. Vinculin served as a loading control. Quantitation of the ratios of TGFβ, IL17, IL6, IL4, IL1β and TNFα to vinculin on the western blots are presented beneath the blots. Data are analyzed by Student’s T-test and presented as mean ± SEM. n = 3 animals per group.

Supplementary Figure S4 Examination of inflammatory factors in the pig’s peripheral tissues after intravenous injection of saline or AAV-GFP. **(A,C)** Western blotting of the heart, muscle, spleen, testis, liver, lung, kidney of saline-or AAV GFP-injected pigs with antibodies against TGFβ, IL17, IL6, IL4, IL1β and TNFα. Vinculin served as a loading control. **(B,D)** Quantitation of the ratios of TGFβ, IL17, IL6, IL4, IL1β or TNFα to vinculin on the western blots. Data are analyzed by student’s T-test and presented as mean ± SEM. n = 3 animals per group.

Supplementary Figure S5 Heat map of correlation of expression levels between WT and GFP.
