# Supplementary material for: Intravenous AAV9 administration results in safe and widespread distribution of transgene in the brain of mini-pig
Source: Front Cell Dev Biol. 2023 Jan 24;10:1115348. doi: 10.3389/fcell.2022.1115348 (PMC9902950; doi:10.3389/fcell.2022.1115348)
Supplement: Supplementary file 1 [file Table1.DOCX]

Supplementary Material

Intravenous AAV9 administration results in safe and widespread distribution of transgene in the mini-pig

Yingqi Lin^1^†, Caijuan Li^1^†, Wei Wang^1^, Jiawei Li^1^, Chunhui Huang^1^, Xiao Zheng^1^, Zhaoming Liu^2^, Xichen Song^1^, Yizhi Chen^1^, Jiale Gao^1^, Jianhao Wu^1^, Jiaxi Wu^1^, Zhuchi Tu^1^, Liangxue Lai^2^, Xiao-Jiang Li^1^, Shihua Li^1*^, Sen Yan^1*^

^1^Guangdong Key Laboratory of Non-human Primate Research, Guangdong-Hongkong-Macau Institute of CNS Regeneration, Jinan University, Guangzhou, 510632, China

^2^Key Laboratory of Regenerative Biology, South China Institute for Stem Cell, Biology and Regenerative Medicine, Guangzhou Institutes of Biomedicine and Health, Chinese Academy of Sciences, 510530 Guangzhou, China

† These authors contributed equally to this work and share first authorship

*** Correspondence:**Corresponding Authors
[lishihualis@jnu.edu.cn](mailto:lishihualis@jnu.edu.cn); [231yansen@163.com](mailto:231yansen@163.com)

1. **Supplementary Figures**
2. **Supplementary Table**

**Supplemental Figure 1.**


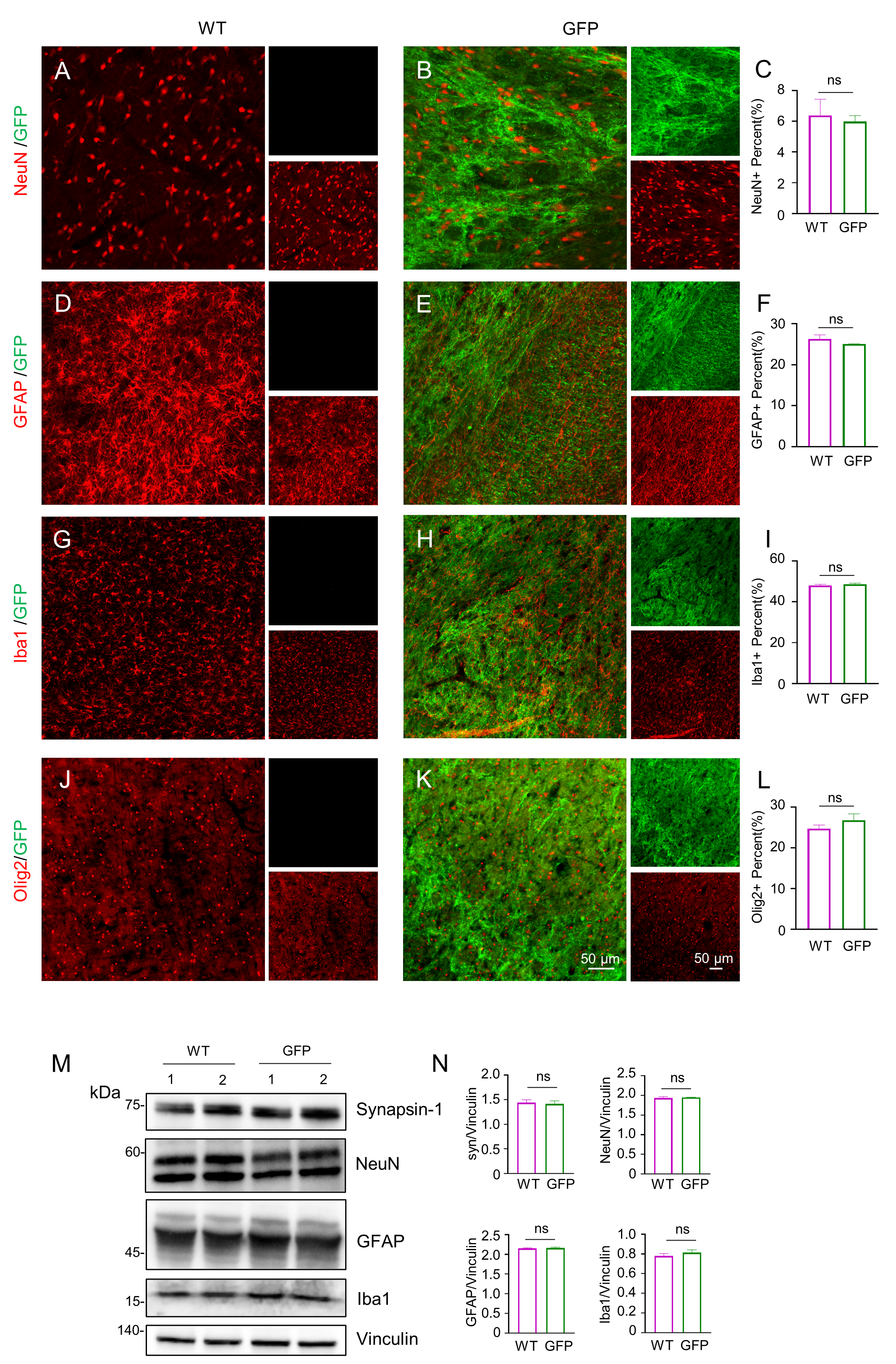


**Figure S1. Immunofluorescent staining of the pig’s brain stem injected with AAV-GFP or saline.**

**(A-L)** Double immunofluorescent labeling and quantification of neurons (NeuN) (A-C), astrocytes (GFAP) (D-F), microglial (Iba1) (G-I) and oligodendrocytes (Olig2) (J-L) of saline- or AAV-GFP-injected-wild type pigs. GFP positive cells are shown in green, NeuN, GFAP, Iba1 and Olig2 positive cells are shown in red. Data are analyzed by Student’s T-test and presented as mean ± SEM. n=3 animals per group. **(M)** Western blotting of the brain stem of saline- or AAV-GFP-injected pigs with antibodies against synapsin-1, NeuN, GFAP and Iba1. Vinculin served as a loading control. **(N)** Quantitation of the ratios of synapsin-1, NeuN, GFAP or Iba1 to vinculin on the western blots. Data are analyzed by student’s T-test and presented as mean ± SEM. n=3 animals per group.

**Supplemental Figure 2.**


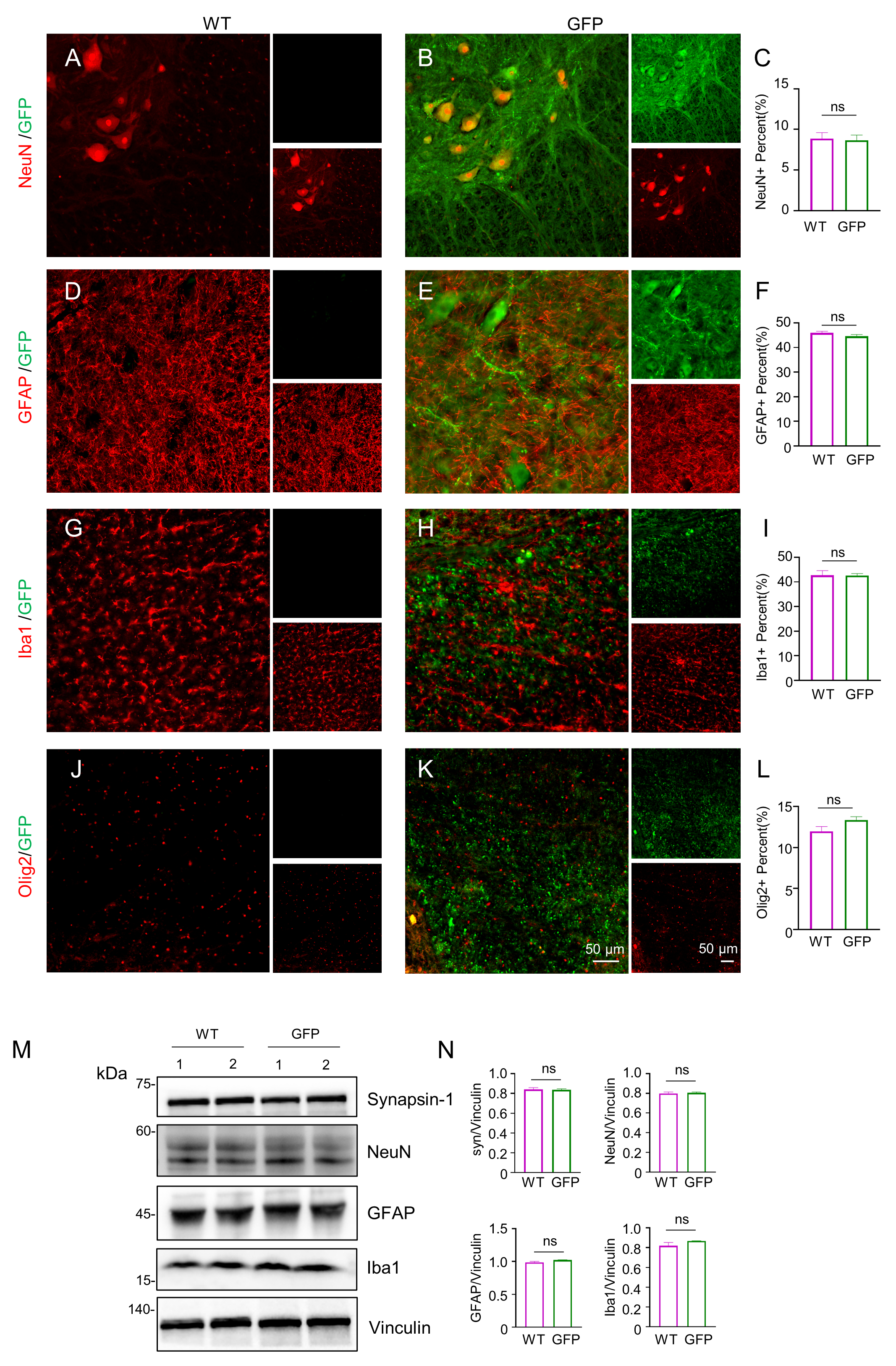


**Figure S2. Immunofluorescent staining of the pig’s spinal cord injected with AAV-GFP or saline.**

**(A-L)** Double immunofluorescent labeling and quantification of neurons (NeuN) (A-C), astrocytes (GFAP) (D-F), microglial (Iba1) (G-I) and oligodendrocytes (Olig2) (J-L) of saline- or AAV-GFP-injected-wild type pigs. GFP positive cells are shown in green, NeuN, GFAP, Iba1 and Olig2 positive cells are shown in red. Data are analyzed by Student’s T-test and presented as mean ± SEM. n=3 animals per group. **(M)** Western blotting of the spinal cord of saline- or AAV-GFP-injected pigs with antibodies against synapsin-1, NeuN, GFAP and Iba1. Vinculin served as a loading control. **(N)** Quantitation of the ratios of synapsin-1, NeuN, GFAP or Iba1 to vinculin on the western blots. Data are analyzed by student’s T-test and presented as mean ± SEM. n=3 animals per group.

**Supplemental Figure 3.**


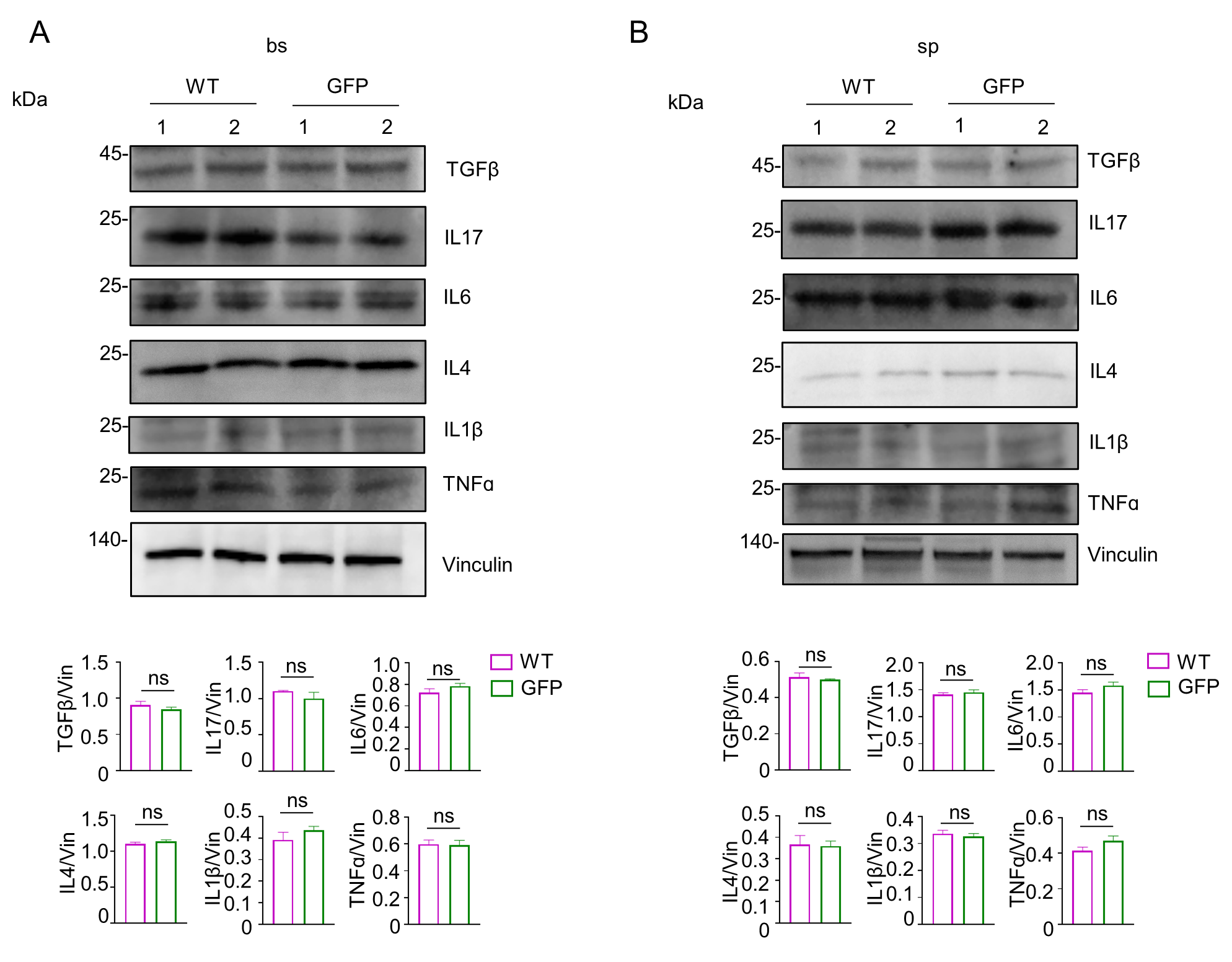


**Figure S3. Examination of inflammatory factors in the pig's brain stem and spinal cord after intravenous injection of saline or AAV-GFP.**

**(A-B)** Western blotting of the brain stem (A) and spinal cord (B) of saline-or AAV GFP-injected pigs with antibodies against TGFβ, IL17, IL6, IL4, IL1β and TNFɑ. Vinculin served as a loading control. Quantitation of the ratios of TGFβ, IL17, IL6, IL4, IL1β and TNFɑ to vinculin on the western blots are presented beneath the blots. Data are analyzed by Student’s T-test and presented as mean ± SEM. n=3 animals per group.

**Supplemental Figure 4.**


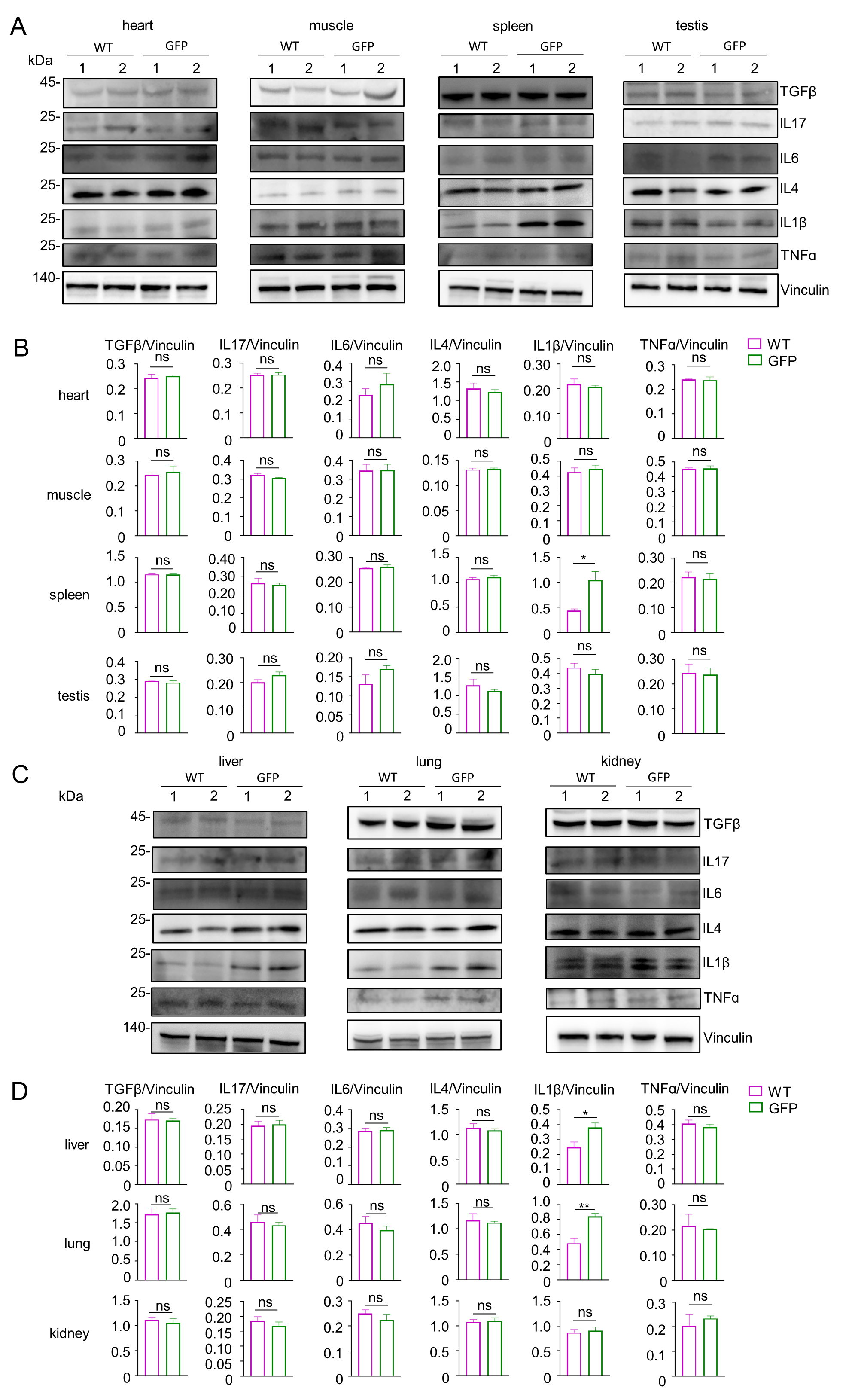


**Figure S4. Examination of inflammatory factors in the pig's peripheral tissues after intravenous injection of saline or AAV-GFP.**

**(A.C)** Western blotting of the heart, muscle, spleen, testis, liver, lung, kidney of saline-or AAV GFP-injected pigs with antibodies against TGFβ, IL17, IL6, IL4, IL1β and TNFɑ. Vinculin served as a loading control. **(B.D)** Quantitation of the ratios of TGFβ, IL17, IL6, IL4, IL1β or TNFɑ to vinculin on the western blots. Data are analyzed by student’s T-test and presented as mean ± SEM. n=3 animals per group.

**Supplemental Figure 5.**


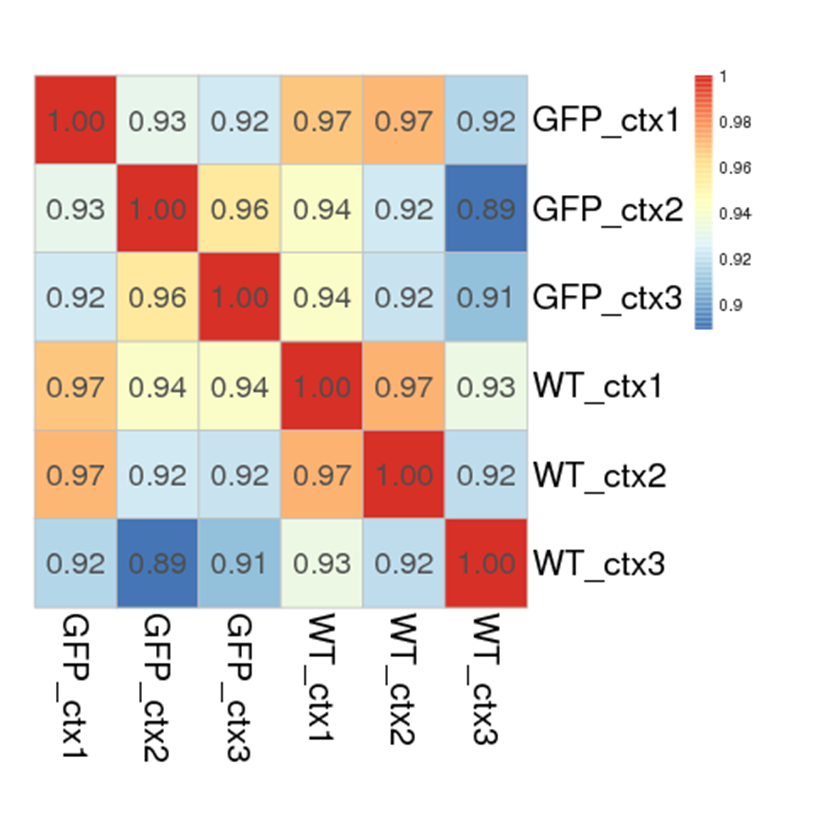


**Figure S5.** **Heat map of correlation of expression levels between WT and GFP.**

**Supplemental Table 1.**

**Supplemental Table 1.** Three brain regions were chosen (cortex, striatum and hippocampus) for qualitative rating of cell type transduction. Scale ranges from – to ++++, with – indicating no double labeling of GFP and the cell specific marker and ++++ indicating the highest level of double labeling observed.
