# Supplementary figures and images for: Intravenous AAV9 administration results in safe and widespread distribution of transgene in the brain of mini-pig
Source: Front Cell Dev Biol. 2023 Jan 24;10:1115348. doi: 10.3389/fcell.2022.1115348 (PMC9902950; doi:10.3389/fcell.2022.1115348)

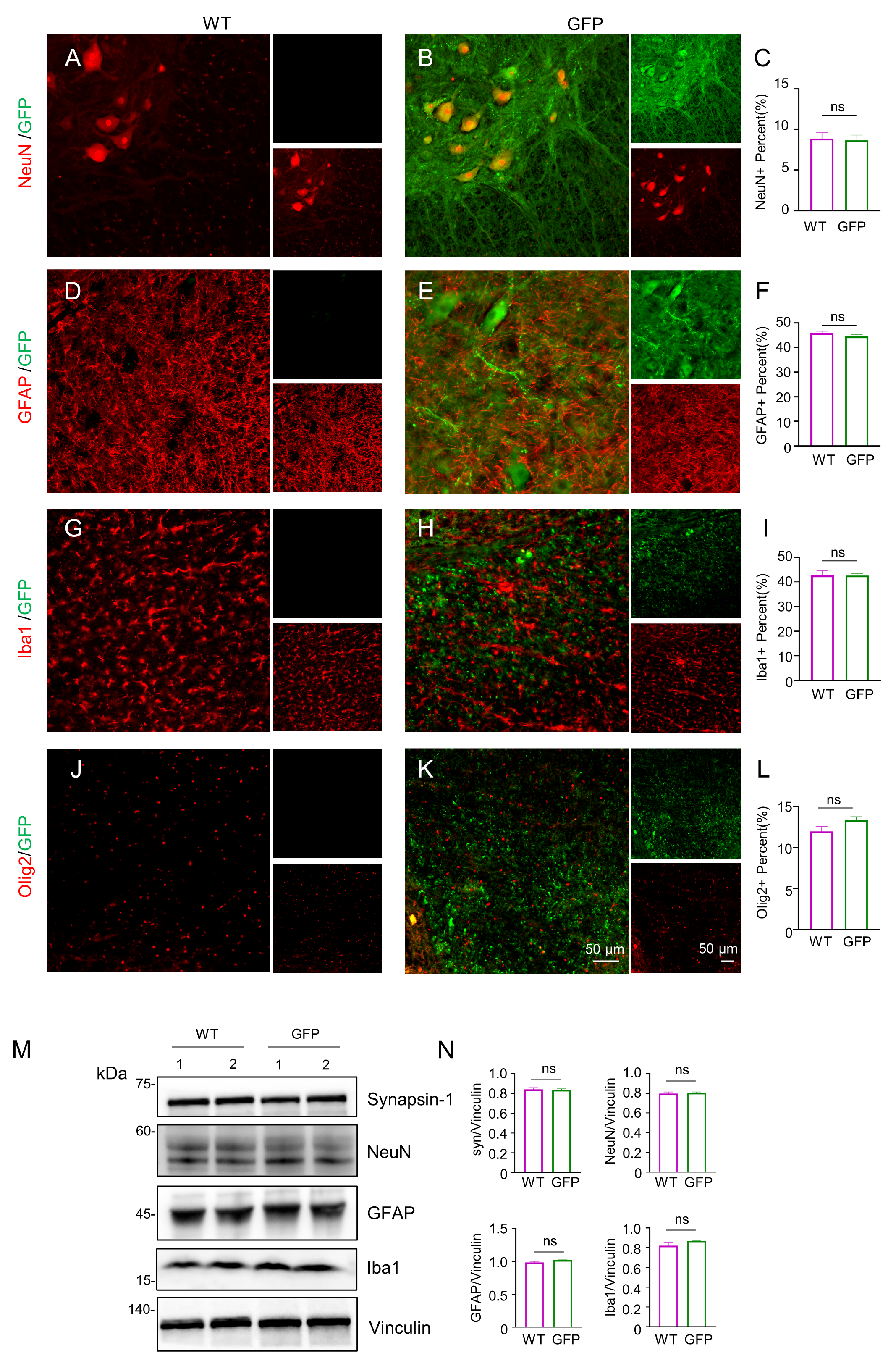

Supplement: Supplementary file 2 [file DataSheet1.ZIP › Supplemental Figure/Supplemental Figure/Supplemental Figure 2.tif]

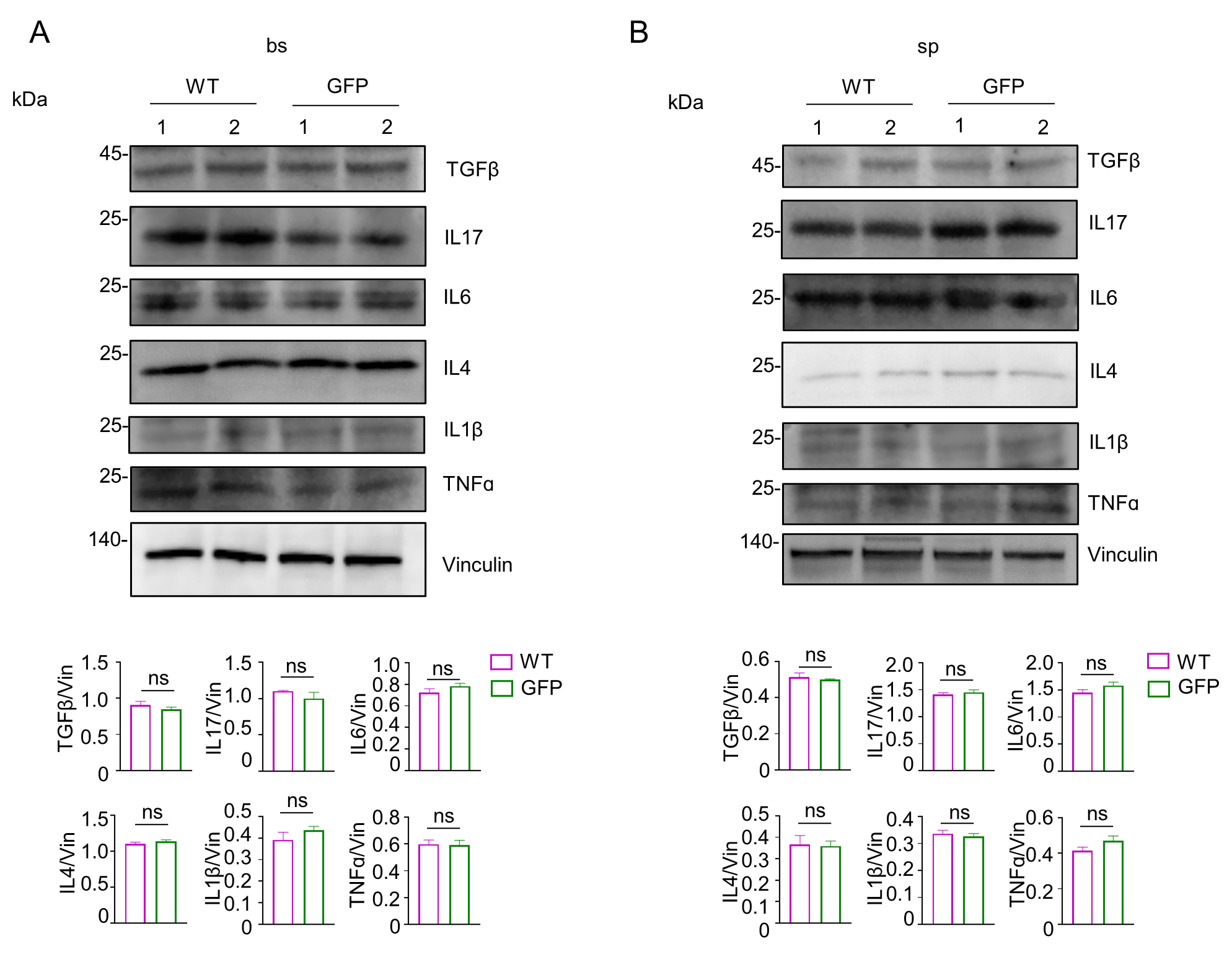

Supplement: Supplementary file 2 [file DataSheet1.ZIP › Supplemental Figure/Supplemental Figure/Supplemental Figure 3.tif]

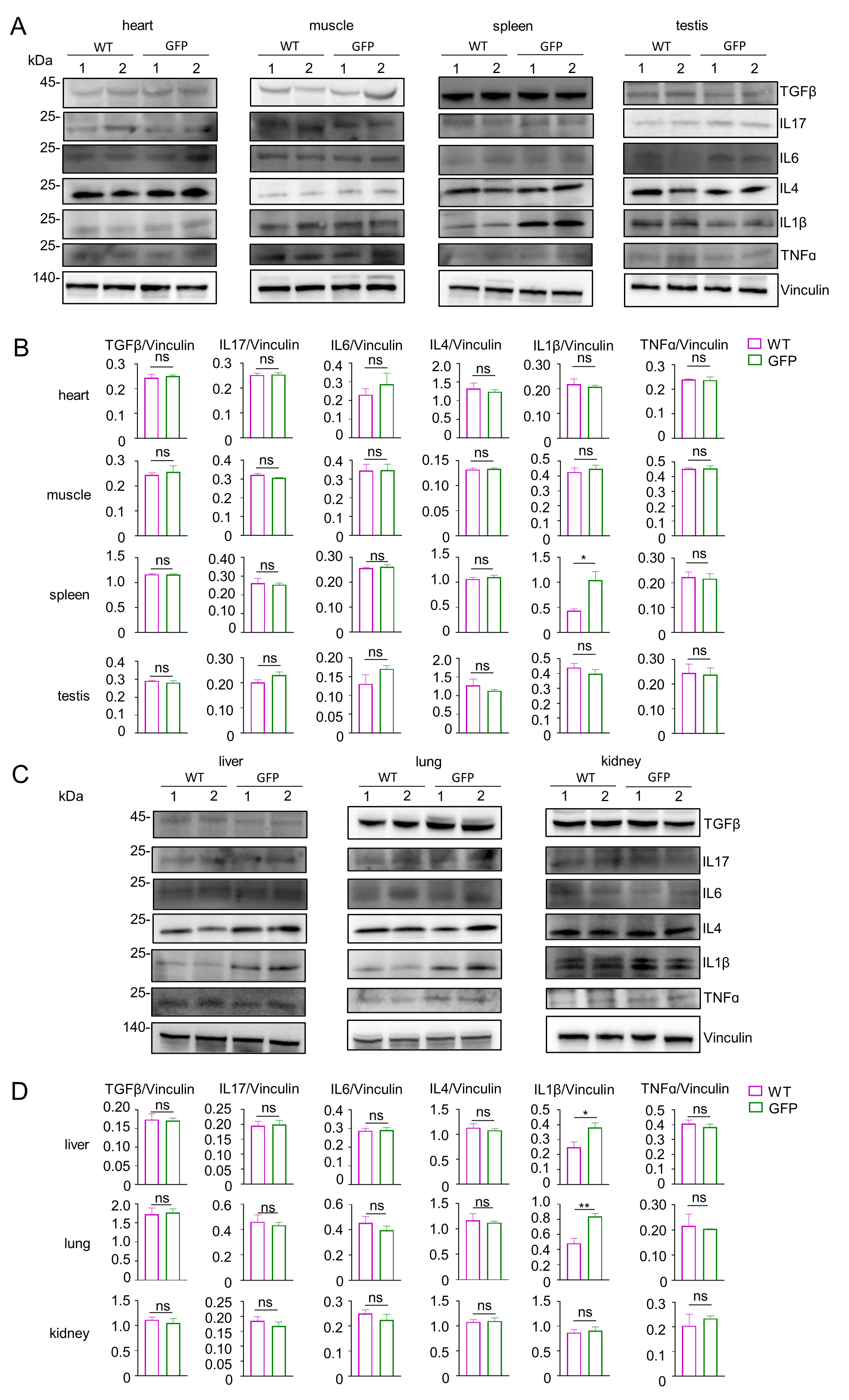

Supplement: Supplementary file 2 [file DataSheet1.ZIP › Supplemental Figure/Supplemental Figure/Supplemental Figure 4.tif]

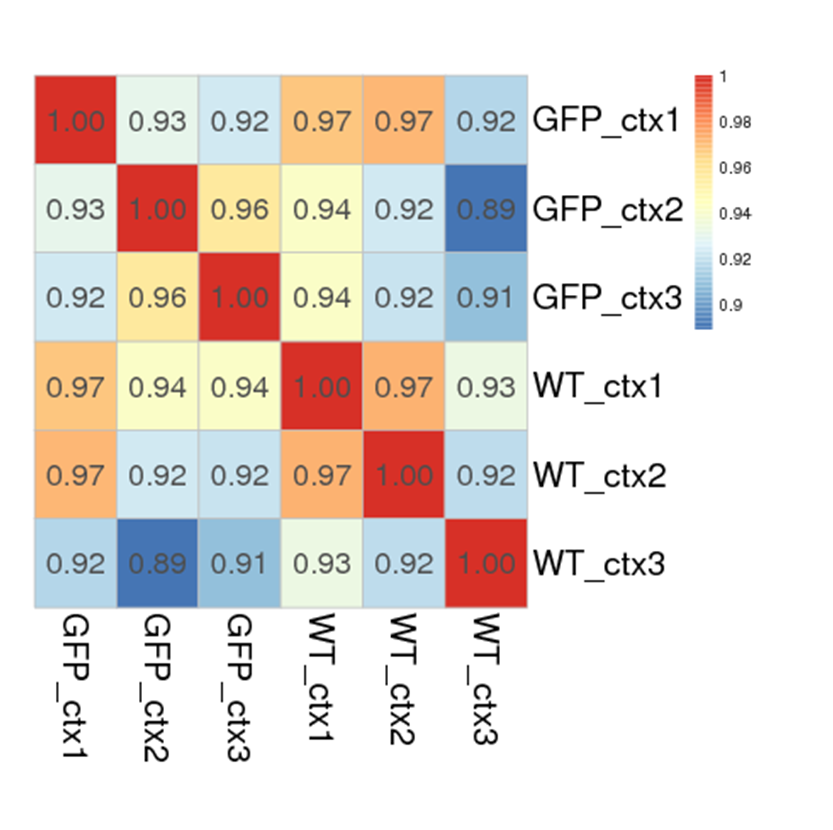

Supplement: Supplementary file 2 [file DataSheet1.ZIP › Supplemental Figure/Supplemental Figure/Supplemental Figure 5.tif]

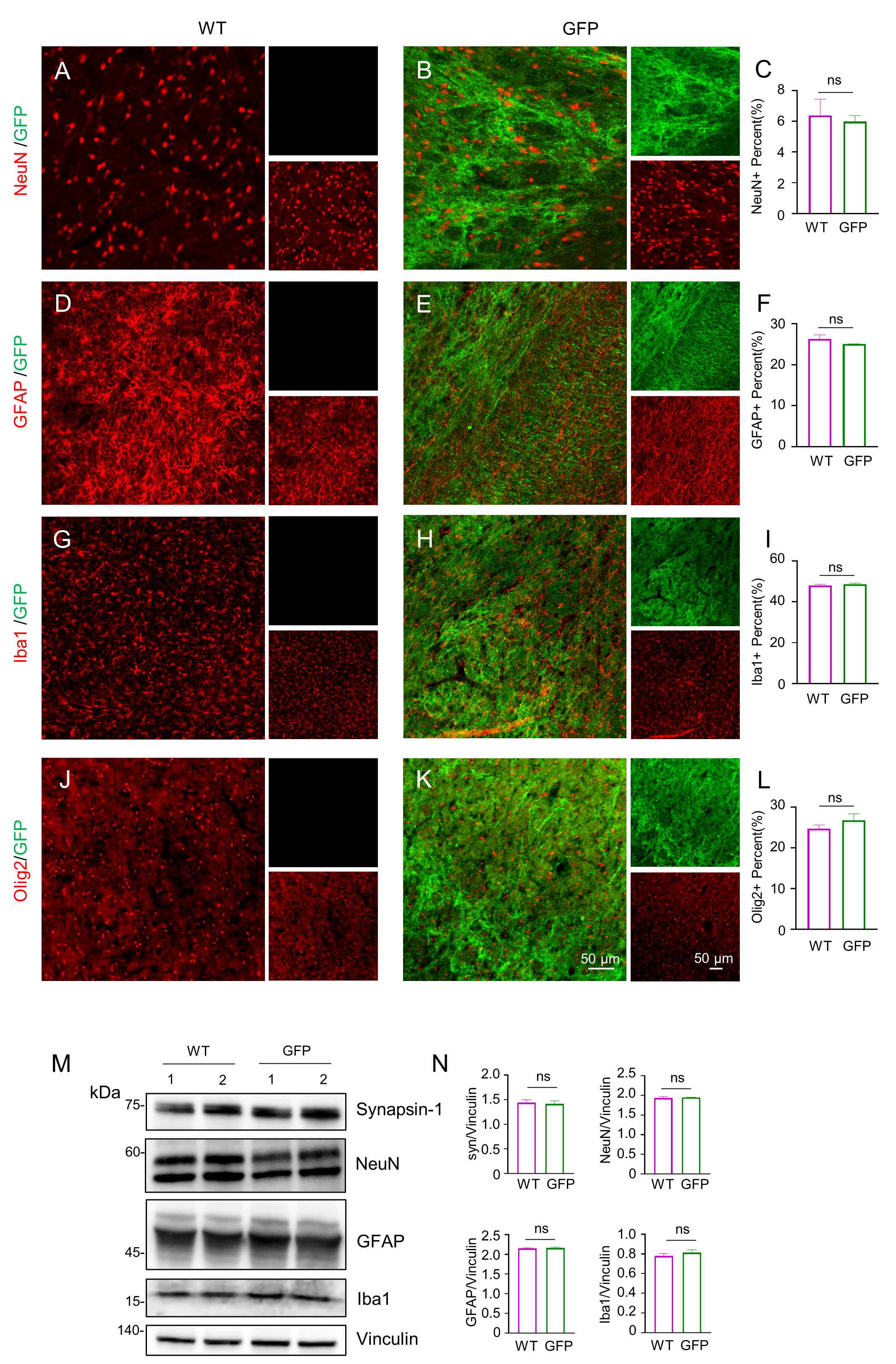

Supplement: Supplementary file 2 [file DataSheet1.ZIP › Supplemental Figure/Supplemental Figure/supplenmental Figure 1.tif]
